# A Clinical Decision Support System for Integrating Tuberculosis and HIV Care in Kenya: A Human-Centered Design Approach

**DOI:** 10.1371/journal.pone.0103205

**Published:** 2014-08-29

**Authors:** Caricia Catalani, Eric Green, Philip Owiti, Aggrey Keny, Lameck Diero, Ada Yeung, Dennis Israelski, Paul Biondich

**Affiliations:** 1 Innovative Support to Emergency, Disease, & Disaster (InSTEDD), Sunnyvale, California, United States of America; 2 School of Public Health, University of California, Berkeley, California, United States of America; 3 Kijani Consulting, Chapel Hill, North Carolina, United States of America; 4 Duke University, Chapel Hill, North Carolina, United States of America; 5 Academic Model for the Prevention and Treatment of HIV (AMPATH), Eldoret, Kenya; 6 Regenstrief Institute, Indiana University, Indianapolis, Indiana, United States of America; 7 School of Medicine, Stanford University, Palo Alto, California, United States of America; US Army Engineer Research and Development Center, United States of America

## Abstract

With the aim of integrating HIV and tuberculosis care in rural Kenya, a team of researchers, clinicians, and technologists used the human-centered design approach to facilitate design, development, and deployment processes of new patient-specific TB clinical decision support system for medical providers. In Kenya, approximately 1.6 million people are living with HIV and have a 20-times higher risk of dying of tuberculosis. Although tuberculosis prevention and treatment medication is widely available, proven to save lives, and prioritized by the World Health Organization, ensuring that it reaches the most vulnerable communities remains challenging. Human-centered design, used in the fields of industrial design and information technology for decades, is an approach to improving the effectiveness and impact of innovations that has been scarcely used in the health field. Using this approach, our team followed a 3-step process, involving mixed methods assessment to (1) understand the situation through the collection and analysis of site observation sessions and key informant interviews; (2) develop a new clinical decision support system through iterative prototyping, end-user engagement, and usability testing; and, (3) implement and evaluate the system across 24 clinics in rural West Kenya. Through the application of this approach, we found that human-centered design facilitated the process of digital innovation in a complex and resource-constrained context.

## Introduction

### HIV and Tuberculosis in Kenya

Around the world, people living with HIV have a 20-fold higher risk than people without HIV of dying from tuberculosis (TB) [1]. Active TB disease can occur at any stage of HIV infection [2], [3] and, as such, routine screening for TB during HIV care provides important opportunities to prevent, diagnose, and promptly treat the disease. Given the vulnerability of people living with HIV, prevention is especially important. A growing body of literature suggests that isoniazid preventive therapy (IPT) reduces overall TB incidence and is therefore of considerable benefit to patients and the larger community [4]. For these reasons, the World Health Organization recommends routine, repeated clinical screening for active TB disease among all people living with HIV and the provision of either treatment for active disease or IPT for asymptomatic patients to mitigate the risk of developing active TB [5]. In broad agreement with WHO recommendations, national governments worldwide, including Kenya, have adopted this as a national health priority, created country plans of action, and produced treatment protocols for clinicians [6]. During recent years, the global health funders have committed significant resources to making TB prevention and treatment medications widely available worldwide [7].

Although the science, financial support, and political willingness to solve this problem are primed, the rate of new TB infections and TB-related mortality continue to increase worldwide due to other complex barriers [8]–[21]. In 2009, of the estimated 33 million people living with HIV worldwide, only 1.7 million (5%) were screened for TB, and about 85,000 (0.2%) were offered IPT [5]. To bring TB prevention and treatment to the world's underserved communities, already over-burdened HIV care systems will need to adapt a more effective means of triaging and monitoring people living with HIV.

In Kenya, approximately 1.6 million people are living with HIV [22]. The AMPATH consortium – a partnership between Indiana University in the United States, Moi University School of Medicine in Kenya, and several other medical schools– has been working to provide support to communities affected by HIV since 1988. A USAID-supported initiative, AMPATH addresses food and income security needs, delivers and monitors antiretroviral treatment, and fosters prevention of HIV transmission.

The HIV burden in AMPATH's catchment is greater than 15% in some areas and, as such, represents the highest incidence of HIV in Kenya. To support HIV care at this scale, the AMPATH system has a network of 49 active clinics across Western Kenya, which collectively manages approximately 40,000 HIV patient visits per month. AMPATH was one of the first clinical care networks in Africa to use an electronic medical record system. The record system first developed and deployed at AMPATH became known as OpenMRS, now the world's most popular open source electronic medical record system with implementations in over 40 countries across every continent [23]–[27]. The availability of digital patient data through OpenMRS has made several other eHealth interventions possible at AMPATH, including an HIV clinical decision support system proven to improve adult and pediatric HIV care [28], [29].

AMPATH leadership had long been committed to TB prevention and treatment but, like the rest of the global health world, struggled to provide IPT to eligible patients. A recent report by Carter et al (2012) described tuberculosis status and services at AMPATH [30]. In it, they find that tuberculosis risk within AMPATH communities is substantial: 23% of adults in the general population have latent TB infection and tuberculosis is the cause of death for one of five HIV patients [30]. Further, they found that 38.1% of all eligible patients initiated IPT and 69.6% of all these patients completed 9 months of IPT during the period of 2004–2008 [30]. However, since 2008, AMPATH's program underwent unexpected challenges as IPT stocks disappeared and providers ceased to initiate IPT and then mostly ceased to screen patients for IPT eligibility.

The TB Tech project was initiated in January 2013 as a catalyst for improving the integration of TB prevention and treatment into HIV services across AMPATH's networks. The TB Tech team identified several levels of potential target groups for intervention – from patients living with HIV to national policymakers – and launched efforts with a focus on HIV clinical care providers within the AMPATH system. Through a human-centered design process, we explored, refined, and delivered an innovative approach to improving the percentage of eligible HIV patients starting IPT.

### Human-Centered Design

Human-centered design (HCD), or user-centered design as it is also known, is a process for facilitating innovation that has been used by industrial and technology designers for decades [31]. It is a process for gaining insight into the needs of the beneficiaries of your innovation, creating innovative approaches to meet these needs, and delivering solutions that work in specific socioeconomic contexts [32]. HCD can be characterized as a multi-stage problem-solving process that requires designers to assess how users are likely to use a product and to test products in real world scenarios with actual users. The International Standards Organization (ISO) standard describes key principles of human-centered design:

- The design is driven and refined by user-centered evaluation;
- The process is iterative;
- The design addresses the whole user experience; and,
- The design team includes multidisciplinary skills and perspectives [33].

There are several specific approaches to HCD that have gained notoriety in the past ten years, including: cooperative design, participatory design, and contextual design. These approaches emphasize particular aspects of the design process, such as equitable engagement of end-users during all stages of the design process for participatory design, but all share the common principles of HCD listed above. Although HCD is not explicitly a research methodology, it incorporates a series of mixed qualitative and quantitative methods to achieve design objectives.

For health technology, the underlying philosophy of the HCD encourages leaders and managers of health innovations to design technology around how users such as clinicians, patients, and community beneficiaries can, want, or need to use technology, rather than requiring them to majorly alter their behavior or attitudes to accommodate the technology. Ultimately, the aim of this approach is to enhance the usefulness, usability, and use of health technology so that health outcomes and impacts can be improved. HCD was chosen to provide insights into innovative technological solutions to address HIV and TB integration in Kenya, a challenge embedded within a complex socioeconomic, medical, and technical context.

### Human Centered Design for TB/HIV Technology in Kenya

The TB Tech project used the IDEO approach to HCD [34] that involves three stages: hear, create, and deliver. To meet the research objectives of each stage, the team utilized a mix of qualitative and quantitative methods as summarized in Table 1 and described in detail in the methods section below.

**Table 1:** Human Centered Design Stages & Research Methods.

HCD Phase	Method	n	Data Type	Data Analysis
Hear	Site observations	9 sites	Qualitative field notes	Grounded theory using Dedoose software (2014)
Hear	Key informant interviews	24 key informants	Qualitative interview audio recordings	Grounded theory using Dedoose software (2014)
Create	Lab simulation testing	217 pseudo patients	Quantitative data reports	Simple descriptive statistics using Excel software (2008)
Create	Clinical usability testing	9 clinicians	Quantitative surveys	Simple descriptive statistics using Excel software (2008)
			Qualitative interview audio recordings	Grounded theory using Dedoose software (2014)
Deliver	Impact evaluation	49 clinics	Quantitative medical record data reports	Cluster-level analysis using unpaired t-test to determine statistical significance with 95% confidence intervals via SAS software (2013).

During the hear stage, HCD designers use research methods to understand social context and inspire new solutions [34]. Qualitative methods are especially emphasized as a means to “uncover deeply held needs, desires, and aspirations” and “analyze and map the relational dynamics between people, places, objects, and institutions” (pp 33). For the TB Tech research team, this entailed conducting site observation sessions and key informant interviews to gather in-depth insights into HIV and TB care within the AMPATH system, barriers to IPT initiation, the electronic medical record and other data systems, and existing interventions to influence provider's care practices.

Next, during the create stage of HCD, designers move from gathering a broad understanding of the problem to real-world solutions. This is accomplished through a process of synthesizing knowledge, interpreting findings into high-level insights and frameworks, and distilling an array of potential solutions into iteratively refined prototypes. During this stage of the TB Tech project, a team of Kenyan clinicians, information technology engineers, and health researchers collaborated over 6 months to translate the insights derived from the hear stage and the experiential knowledge of Kenyan clinicians into the design of a TB clinical decision support system. Specifically, the team developed detailed TB treatment protocols, computer-based algorithmic rules for the clinical decision support system, and health communications guidelines for the decision support message that providers would receive. Using these protocols, rules, and guidelines, the TB Tech team produced several prototype decision support systems and worked closely with end-users to iteratively develop prototypes. Two research methods were used to test these prototypes: lab simulation and in-context usability testing.

Finally, the TB Tech team worked to rollout solutions to constituents during the deliver stage. As of March 2014, the TB Tech team rolled out the beta version, second major iteration, of the clinical decision support system across a network of 68 clinics in Western Kenya. Critical to this process, as IDEO [32] describes it, is “on-going measurement, evaluation, and iteration” to ensure that “the solutions developed stay grounded in real-world impact and continue to evolve” (pp 125). To address these objectives, the TB Tech team is currently leading an impact evaluation to measure its effect on the integration of TB and HIV care. The evaluation protocol is described elsewhere [34] and findings will be reported in an ensuing publication.

This study explores the use of the HCD approach and relevant research methods for understanding the problem of tuberculosis among people living with HIV, creating an innovative system for improving the TB prevention and treatment practices among HIV providers, and deploying a solution in clinics across rural western Kenya.

## Methods

As described, the HCD approach involves strategic use of qualitative and quantitative research methods to meet the objectives of each stage of design. For the TB Tech initiative, this included site observations, key informant interviews, lab simulation, and in-context usability testing.

### Data Collection

#### Site Observations

The TB Tech research and clinician teams identified 9 sites for visit and observation sessions, among the 49 active sites in AMPATH's networks. These sites were purposefully selected with the aim of diversity across the following key characteristics: rural/urban, average monthly patient volume, total number of active providers, and AMPATH's clinical categorization of 1–6 (1 being a village kiosk and 6 being a district referral hospital). Site observations required 3–4 hours each and were conducted by a trained ethnographic researcher, using a semi-structured observation guide. Site observation field notes were used in analysis.

#### Key Informant Interviews

The TB Tech team identified key informants through purposeful selection with the aim of diversity across the following key characteristics: health area of expertise (HIV, TB, information technology), role at AMPATH (clinical, managerial, research), department (laboratory, pharmacy, radiology, clinic), and geographical responsibility (regional office, mother site, satellite site, village home-to-home). Key informant interviews required 45 min to 1 hour and were conducted by two trained qualitative researchers using an unstructured interview guide. Interviewees included medical superintendents, clinicians, Ministry of Health officials, laboratory managers, pharmacy managers, medical directors, TB care providers, AMPATH administrators and program managers, data quality workers, and community health workers. During the interviewers, one researcher facilitated the interview while the recorded audio and took detailed notes. All interviews were conducted in English. Audio recordings were used in analysis.

#### Lab Simulation Testing

Using a set of 217 pseudo-patients, the TB Tech programming and development team ran the clinical decision support system algorithms and identified the message content that would be delivered to a clinician during that particular patient's visit. Pseudo-patients were mock patients whose histories and electronic medical records were based on actual patients. Lab simulation allowed the TB Tech team to assess the system's accuracy. Clinical accuracy was based on AMPATH's standards and priorities for TB prevention and treatment. During this iterative process, any time that the decision support system produced an inaccurate message or had a missing message, the system rules were refined and lab simulation was repeated. This process was repeated until all pseudo patients received the medically appropriate decision support message. Quantitative reports with the details of the number of false negatives, false positives, and correct messages occurring during each iteration of lab testing were used in analysis.

#### In-Context Usability Testing

The TB Tech team conducted usability testing at three clinical sites, among ten HIV clinicians. These sites were chosen based on convenience, as the clinical leadership at each site was open and interested in testing an innovative approach to TB care integration. During usability testing, a mixed-methods usability survey was used to assess clinician's perceptions of the understandability, importance, helpfulness, practicality/feasibility, and accuracy of each TB reminder message that they received throughout a normal day of clinical visits. Clinicians scored reminders on a scale from 1 (very bad) to 5 (very good) for these five usability criteria. These quantitative survey responses were used in analysis. At the end of a day clinical visits and experience with the new system, clinicians participated in in-depth interviews to provide feedback on the system as well as the general challenges and opportunities for TB and HIV care integration through clinical decision support and other interventions. Interviews were conducted by two trained interviewers. Interviews required 20–30 minutes each, were conducted in English, and were audio recorded using a Livescribe Sky digital pen. Qualitative audio recordings of interviews were used in analysis.

### Data Analysis

All qualitative data, including key informant interview recordings, site observation field notes, and in-context usability interview audio recordings, were analyzed using a modified ground theory approach [36]. This approach involved identifying codes from within the data, systematically applying codes to data, and extrapolating to a set of thematic constructs relevant to opportunities and challenges for the integration of HIV and TB care. Coding and analysis of qualitative data, including direct coding of audio files, was facilitated by Dedoose version 4.5.95 copyright 2014, a cloud-based mixed methods analysis software.

Quantitative data collected during the formative stages of our HCD initiative, including lab simulation reports and in-context usability surveys were analyzed using simple descriptive statistics through Microsoft Excel version 12.3.6 copyright 2007.

### Ethics Statement

This study was approved by the ethics review committees at institutional partners Population Council, Indiana University and Moi University in Kenya. The study relied exclusively on de-identified data, meaning that the site observation field notes, interview transcripts, and ratings forms included no documentation of names, geographical location, contact information, personal identification numbers, descriptions of physically identifiable characteristics, or other established identifiers. As such, all three IRB committees determined that consent was unnecessary. Population Council's IRB determined that the study did not actually involve “human subjects” was exempt. Nevertheless, as a courtesy to those participating in key informant interviews, our team obtained oral consent by reading a short statement about the risks and benefits of voluntary participation. If key informants volunteered to participate, then the interview team checked a box on the interview guide indicating as such.

## Findings

During each stage of the HCD process, research data was analyzed and findings were reported to the team. As such, findings were consistently integrated into the iterative process of refining the team's understanding of the challenge of integrating HIV and TB care, identifying innovative solutions, developing prototype systems, and deploying a new system.

### Hear

To understand social context and inspire new solutions during the hear phase, researchers conducted site observation sessions and key informant interviews. Analysis of this qualitative data revealed four major themes relevant to the challenges and opportunities for TB and HIV integration: clinician's attitudes about IPT, clinician's knowledge of IPT, clinician's practices around IPT, clinician's perceptions of information systems, and clinical resources for IPT case-finding.

#### Clinician attitudes about IPT

Through interview and observation sessions, some key attitudes about TB screening, TB testing and diagnosis, IPT initiation, and TB/IPT treatment and adherence became evident. Clinicians believed in the importance of identifying HIV patients who are eligible for IPT, however they reported several major barriers to initiating IPT.

First, they reported that it was often difficult to manage TB priorities among many other priorities. They explained that, in this clinical setting, all walk-in and scheduled patients arrive early in the morning and form a line to see providers. Providers see patients according to the order in line and reported some pressure to see as many patients as possible, as quickly as possible. On a typical day, a single provider might see 20–40 patients before they break for lunch. Frequently, providers explained that managing just HIV care is quite demanding, although they have substantially more training, experience, and institutional resources for this care. Patients may not see the same provider from visit to visit and so providers explained that they rely on a patchwork of paperwork and patient self-report to determine what steps in intensified TB case-finding and IPT eligibility have been completed.

Secondly, and most commonly reported among clinicians, in the rare case in which IPT eligibility could be confirmed during a patient visit, providers remained hesitant to start a patient on IPT due to pharmacy shortages. Previous experiences with periodic and long-term “stock-outs,” as participants described them, made clinicians wary of contributing toward drug resistance by exposing patients to IPT and not completing the regimen. And, so, as clinicians repeatedly explained, if they were not confident that all of their eligible patients had a 9-month supply of IPT, they preferred not to start IPT at all.

#### Clinician knowledge of IPT

Interview transcripts and observation sessions alike revealed that, although some clinicians were quite knowledgeable about global and institutional standards for determining IPT eligibility, most had insufficient knowledge to determine eligibility for their patients. In particular, they often described being unsure about the role of chest x-ray, when and if it should be ordered and how to read radiographs. Most providers reported receiving no special training in TB and only some providers reported receiving a two-day training in reading chest radiographs.

#### Clinician perceptions of information systems

Clinicians and managers were overwhelmingly positive about the electronic medical record (EMR) system and, in particular, the HIV clinical decision support summary. Key informants explained that without it, providers would likely forget or overlook critical steps in HIV care. They emphasized that reminders were prompts, simple and shorthand cues related to services that they were already well trained and experienced in providing. Many agreed that a simple prompt about IPT eligibility might not be adequate to answer providers' broader questions and concerns about initiating IPT.

Clinicians perceived the information systems, however helpful, to frequently suffer from missing patient data. They explained that it was common for a clinician to be prompted to provide a service that had already been provided. Information system managers understood that this problem derived, in part, from the lack of integration between patient's EMR, laboratory management information systems, pharmacy information systems, radiology information systems, and TB patient records. Given this integration challenge, it is not surprising that clinicians most typical complaint about the patient information systems was missing documentation on chest x-ray orders and results.

#### Clinical resources for determining IPT eligibility

Clinicians and facility managers explained that there were several clinical resources needed to rule out TB among patients who exhibited some potential symptoms such as cough, night sweats, fever, and weight loss. Often, laboratory analysis of sputum samples was frequently necessary. TB testing and diagnostics services were available at nearly every site, even those located in more rural settings. The more accurate, timely, and expensive GeneXpert diagnostic test was available at centralized AMPATH sites, but was reserved for diagnosis among a subset of complex cases. Additionally, and as described above, AMPATH clinics require a chest x-ray to determine patient eligibility for IPT. Chest x-ray facilities, however, were centralized and not available at most smaller and satellite sites. Interviews and observations confirmed that radiology capacity varied during any given week, with unpredictable downtimes due to equipment, electricity, and staffing challenges. Managers at AMPATH described intensive institutional efforts to ensure x-ray access to all patients, including building and deploying mobile x-ray vehicles, but acknowledged that access remains a challenge.

### Create

As the team developed and refined a clinical decision support system to integrate HIV and TB care during the create stage, findings from lab simulations and in-context usability testing were immediate translated into prototypes.

#### Lab simulation

Laboratory simulations with pseudo-patients revealed challenges in developing a clinically accurate decision support system. During each iteration of the system's algorithm, based on computer-based IPT eligibility rules and the ensuing programming code, laboratory simulation identified cases in which patients wrongfully received a particular reminder or wrongfully received no reminder at all. The objective of these simulations was not to identify detailed sensitivity and specificity ratios, but rather continue to improve the design until there was no evidence of errors. The team conducted dozens of cycles of prototype development and simulation before reaching zero errors.

#### Usability testing in context

During the development of alpha and beta versions of the clinical decision support prototype system, lab simulation was followed by in-context usability testing for a small-scale assessment of how the system performed in real world contexts. Usability criteria and clinicians' ratings of IPT reminders received during a day of typical patient visits are described in Tables 2–3. Providers rated all reminders that they received, yielding a total of 51 reminder ratings. Ratings provided usability insights into the IPT clinical decision support system's data (the patient EMR data), algorithm (the computer-based rules and programming), content (the written messages), and medium of delivery (the paper clinical summary sheet).

**Table 2:** Message content ratings.

Criterion	Range (n = 51 observations)	Mean (n = 51 observations)
Understandability	1–5	4.4
Importance	1–5	4.5
Helpfulness	1–5	4.3
Practicality/feasibility	1–5	4.2

**Table 3:** Accuracy and actionability.

Provider prompts used in in-context usability surveys	Yes % (n = 51)	No % (n = 51)	Not sure % (n = 51)
Decision support message was correct for this patient today	**51.0%**	**27.5%**	**21.6%**
I was able to take the next step recommended for TB care today	**33.3%**	**45.1%**	**21.6%**

The understandability, importance, helpfulness, and practicality/feasibility of IPT messages all ranked highly, ranging from 4.2 to 4.5 on a scale of 1–5. Although providers rated the messages relatively highly, they found the accuracy and actionability of the clinical decision support system problematic. Providers indicated that roughly over a quarter of the reminders were not correct for that particular patient and that particular day. Moreover, slightly less than half of the reminders were not considered actionable on that day.

In-depth interviews with providers at the end of a day of receiving IPT messages revealed some of the reasoning for these ratings and assessments. Most of the messages deemed incorrect were related to ordering a chest x-ray. Providers explained that the EMR was missing chest x-ray data although other information, such as paper documentation in the patient record or patient self-report, indicated that the chest x-ray had in fact been ordered and/or read. One provider explained,

“If I see the chest x-ray missing, first I ask the patient if they did it recently. If they say yes, then I ask what the result was. If they tell me that everything was ok, then I make a note of it to update the information. And sometimes I repeat this note to the data team several times and nothing changes in the system. I keep getting the reminder.”

When probed, clinicians revealed that they often based diagnosis and care decisions on patient self-report. They ask patients for their past diagnoses, testing results, and other patient history details, and if this information conflicts with the EMR history, they frequently trust patient recall over the EMR data.

Clinicians reported that they had little special education or training around TB care and were unsure about the steps for determining IPT eligibility. More than just alerts or reminders about steps missed in the past, clinicians requested proactive information about what actions to take moving forward. For example, one clinician asked:

*“So, it says to check for chest x-ray. And what if I find the results now? Then what do they want me to do? Am I to wait for the next time they come in to find out what I should have done?”*

As such, several providers indicated that they were unable to take the action step recommended for TB care because they were not sure what that next step might be.

Additionally, even when they were aware of the appropriate next step toward initiating IPT, many providers described other barriers or concerns that resulted in not complying with the recommended next steps in care. Hesitance and inability to act were focused in three areas: 1) providers did not share the institutional prioritization of chest x-rays for all HIV patients, regardless of the presence or absence of TB or other pulmonary symptoms; 2) providers were not confident in the sustainability of IPT supplies, in agreement with findings during earlier key stakeholder interviews; and, 3) chest radiography was frequently inaccessible during the same day as the patient visit and referral due to equipment failure and staffing inadequacies.

Findings from lab simulations and in-context usability testing were used to refine the clinical decision support system. By the end of this stage, the team created a system to integrate HIV and TB care through the clinical decision support system. To describe how this system functions, first, the provider writes patient information on a paper encounter format the point-of-care while conducting a patient exam as usual. When computer hardware becomes accessible and acceptable in medical offices, then this information will be entered directly into the patient electronic medical record. Second, a data entry team inputs paper encounter forms into the OpenMRS electronic medical record system. Third, computer-based algorithms for TB and IPT care produce messages that are patient-specific, educational, and promotional to inspire behavior change among HIV providers. The content from these messages is included in table 4, with key characteristics identified. If a required action is not completed, such as most commonly due to delays and breakdowns in radiography, then the reminder is repeated during the next patient visit. Before the patient's next scheduled visit, the decision support messages are delivered to providers through the most reliable and feasible means: paper. This system produces individualized & tailored reminders, to be printed on a clinical summary sheet & placed in the patient paper file—the only means to ensure that these messages are seen exactly when providers need them and in a medium that is acceptable and accessible regardless of access to digital hardware, internet networks, and power at the point-of-care. This design was made feasible and acceptable by relying on many existing AMPATH procedures and systems, clinical resources, and provider practices.

**Table 4:** Tailored, educational, & promotional message content.

Message Objective	Patient-Specific, Educational & Promotional Message Content
**Remind to screen to determine active TB status**	TB symptoms include chronic cough, fever, & weight loss. Please ask [patient name] about all symptoms and order/interpret a CXR. AMPATH is committed to offering anti-TB meds or IPT to all eligible patients.
**Remind to conduct symptomatic screening when CXR normal**	TB symptoms not recorded for [patient name] in last encounter. Patient has NORMAL CXR. If no symptoms, consider initiating IPT. IPT saves lives.
**Remind to conduct symptomatic screening when CXR abnormal**	TB symptoms not recorded for [patient name] in last encounter. Patient has ABNORMAL CXR. If patient has symptoms, consider initiating TB treatment. TB treatment saves lives.
**Remind to conduct ongoing symptomatic screening for patients on IPT**	TB symptoms not recorded for [patient name] in last encounter. AMPATH requires continued screening of patients while on IPT. Symptoms may mean that [she/he] has active TB and needs to stop IPT.
**Remind to obtain CXR to determine active TB status when symptoms suggestive of TB (possible TB treatment initiation)**	[Patient name] reported TB symptoms during the last encounter. Please order CXR to determine if [she/he] has active TB and needs to begin lifesaving treatment.
**Remind to order further investigations when CXR is normal and symptoms present**	[Patient name] reported TB symptoms during the last encounter. [Her/His] CXR results were normal. Please order further tests such as sputum microscopy to rule out TB. TB treatment is free and available.
**Remind to initiate anti-TB meds**	[Patient name] may have TB. [Her/His] reported TB symptoms during the last visit and had an abnormal CXR. Order sputum test to determine if [she/he depending on gender] should start lifesaving TB treatment today.
**Remind to consider stopping IPT**	[Patient name] reported symptoms suggestive of TB at last encounter. Symptoms could mean that [he/she] has active TB and needs to stop IPT.
**Remind to obtain CXR to determine active TB status when symptoms NOT suggestive of TB (possible IPT initiation)**	If patient still does NOT report TB symptoms today, a normal CXR means that [he/she] is eligible for IPT. IPT could save [his/her] life. Order CXR to determine IPT eligibility or record existing results to end this reminder.
**Remind to initiate IPT**	[Patient name]'s test results do NOT suggest active TB. If patient still does not report TB symptoms today, consider initiating IPT now. IPT is effective and could save [his/her] life.
**Remind to order further investigations when CXR is Abnormal and symptoms absent**	[Patient name] reported no TB symptoms during the last encounter, however CXR results were abnormal. Please order further tests such as sputum microscopy to rule out TB. At AMPATH, we are committed to stopping TB.
**Remind to monitor adherence to IPT regimen**	[Patient name]'s adherence to IPT was not reported at the last encounter. Please monitor adherence until the patient completes a 9-month course or stops for other reasons. IPT only saves lives when adherence is high.
**Remind to encourage patient to complete IPT if not adherent**	[Patient name] reported low IPT adherence at the last encounter. Please encourage [her/him] to complete the full 9-month course by discussing barriers to adherence. IPT only saves lives when adherence is high.

### Deliver

During the deliver stage of the TB Tech project, the team assessed and addressed feasibility challenges, prepared to roll-out operations, implemented the clinical decision support system, and commenced a randomized clinical trial to assess impact. In assessing and addressing feasibility challenges, the team identified major stumbling blocks to successful implementation and impact based on findings from formative research described above. These included technology systems requirements such as functional printers, reliable electricity, and improvements to EMR data quality and they also included clinical requirements such as adequate IPT stocks and supply-chain management, improved access to radiology, and provider knowledge of TB and IPT standards of care. Working in close partnership with AMPATH, our team committed six months to addressing feasibility challenges to the extent possible. In doing so, some of the key activities included: purchasing and distributing new hardware and supplies, hiring and training new staff to collect critical TB and IPT data at each site, improving IPT supply chain management through decentralized stockpiles and detailed accounting of stock supplies, strategically locating mobile radiology units in proximity to clinical sites with most limited access, and carrying out a TB and IPT educational campaign among all AMPATH providers and clinics.

There were minimal activities required to prepare for operations rollout. By design, the clinical decision support system would build on existing practices across the AMPATH system. By relying on paper print outs and data from an existing EMR system, no fundamentally new equipment or practices needed to be introduced or managed. Moreover, our staff and efforts were focused on ensuring that the existing system was functioning optimally and that known challenges, as listed above, were addressed adequately. The clinical decision support system was first rolled-out to randomly selected clusters of clinics among 68 active sites, as a part of a randomized clinical trial reported elsewhere [35].

## Discussion

Global donors, national ministries of health, and health leaders worldwide have prioritized the integration of HIV and TB care and, in particular, increasing the number of people living with HIV who have access to life-saving IPT. Despite what seems like the perfect storm of funding, science, and political will, the WHO estimated that only 1 in 500 PLHIV are offered IPT [5]. The TB Tech team used techniques human-centered design principles and practices to understand the challenges and opportunities surrounding TB integration in a low-resource HIV care context, create an innovative clinical decision support system to improve TB intensive case-finding and IPT initiation, and implement the system within an extensive network HIV clinics across Western Kenya.

During the hear stage, the team gathered qualitative and quantitative data through observation sessions and in-depth key stakeholder interviews. Several key themes emerged from this data relating to clinicians' attitudes about IPT, knowledge of IPT, practices around IPT, perceptions of information systems, and resources for IPT initiation. To build, test, and refine prototypes of an clinical decision support system during the create stage, the team partnered with Kenyan HIV/TB clinicians, conducted lab simulations with pseudo-patients, and carried out in-context usability tests. Finally, during the deliver stage, the team identified and addressed major barriers to implementation related to technical infrastructure and human capacity, prepared for the few procedures needed to roll-out operations, implemented the clinical decision support system across dozens of clinics, and commenced a randomized clinical trial to evaluate its impact.

There are several limitations to the particular methodological approach used during this HCD initiative and these must be considered in terms of research rigor. With the exception of the randomized clinical trial, currently underway, most methods relied on purposeful sampling strategies. As such, there may be unknown biases that limit the generalizability of these findings beyond those research participants included in the study. Additionally, reporting bias may be a threat within the interview-derived data, given that employees were asked to assess a program that they perceive to be initiated by their employers. This bias may be toward more positive findings, since some employees may be reluctant to be seen as incompliant or unprepared to embrace innovation. Although using multiple methods to triangulate findings may balance the biases of any one particular method, these issues should still be considered.

Throughout the past twenty years, increasing access to inexpensive, durable, and simple technologies has created an opportunity for innovation in global health [36]. Specifically, computerized clinical decision support systems (CDSSs) used to “aid in the reduction of medical errors and in the reduction of adverse drug events, ensure comprehensive treatment of patient illnesses and conditions, shorten patient length of stay, and decrease expenses over time” [37]. In low-resourced settings, CDSSs provide an opportunity to support the competencies of local health workers and “catalyze decision-making in situations where there is time pressure and no possibility to seek advice from other professional colleagues” [38]. In a recent series of six systematic reviews covering 166 randomized controlled trials, a team of researchers found that CDSSs improved the process of medical care in the majority of the studies [39]–[44]

Nevertheless, there has been is scarce, if any, reporting on best practices in design and how well-tested design practices might improve adoption, usability, costs, and the ultimate outcomes and impacts of global health innovations [38], [44]–[45]. Human-centered design has been described as one of the most well-known international standards [46] for design, however, these standards have not been widely used or even discussed in global health.

Within the design arena, there are two main critical arguments against human-centered design, which are essential to discuss in terms of possible unintended consequences of its use for global health. First, due to its strong emphasis on interacting with specific communities, some contend that products developed through HCD may be poorly equipped to serve large populations. Donald Norman, a well-known author and leader in product design argued that “The more something is tailored for particular likes, dislikes, skills, and needs of a particular target population, the less likely it will be appropriate for others” [47]. The implications are that global health innovations that emerge from HCD processes might only be appropriate for the specific community within which it was designed. As a field that has been widely criticized for its “chronic pilotitis,” or the proliferation of interventions that are limited in size and scope and cannot be delivered sustainably across large populations [48], this is a particularly salient concern.

Although more evidence is required to determine the veracity of this threat, the TB Tech experience is one example of using HCD to introduce innovation across dozens of clinical environments, to impact health services delivered to thousands of patients, in a manner that seems likely to be financially sustainable for the foreseeable future. It could, however, be argued that the TB Tech system might only be duplicated within health systems with some minimum requirements, such as: an electronic medical record system, adequate data quality, and TB treatment and prevention capacity and infrastructure. Certainly, not every clinical system around the globe can meet these requirements. Yet, the TB algorithms, provider messages, and system of delivery may likely be adapted and used in other low-resource clinical settings around the world. Furthermore, even if these solutions are only useful within this specific context, several experts argue that overly generic health information technology solutions are yet to be proven effective in any context [49]–[50].

A second common critique of HCD is that it relies too much on feedback from users who might not fully understand what they want and need. Apple Computer founder and digital innovator Steve Jobs famously stated “A lot of times, people don't know what they want until you show it to them” [51]. Henry Ford is quoted as saying “If I had asked people what they wanted, they would have said faster horses.” The implication of these statements is that global health innovation emerging from HCD processes might be limited in terms of creativity, radical leaps, or its ability to spur fundamentally disruptive change in the way that global health services are delivered.

However, it might also be argued that iconic innovators, like Jobs and Ford, were engaged in creating technologies and experiences for the culture and context in which they had spent their lives. Furthermore, these innovators would continue to living alongside the technologies they created, learning and iterating during entire lifetimes. In global health innovation, as was true for the TB Tech team, many of the key members of an innovation team are not from the cultures and contexts in which they work. Although the TB Tech team included experts in health research, technology development, and global health, neither this expertise nor their personal background were adequate for understanding the needs and capacity of HIV providers working in under-resourced clinics across rural Kenya. The HCD approach encouraged consistent and iterative engagement of key stakeholders and end-users, which facilitated the creation of an innovative approach to TB care that did not require substantial changes to existing clinical practices.

Moreover, the process of engaging stakeholders may be particularly helpful in gaining the trust, buy-in, and permission needed to implement change within a complex health system. Unlike other private enterprises, health systems are comprised dynamic interactions between multiple gatekeepers, stakeholders, and decision-makers from policymakers to funders to providers to patients, all of whom balance different objectives and priorities [52]. Since many health innovations involve introducing a new process, as opposed to a new product, an approach to design that facilitates communication and consensus may be especially helpful.

Advocates of more participatory approaches to design, such as HCD, argue that outsider-driven design can lead to tools and technologies that suffer from adoption and usability [53]–[56]. The consequences of failing innovations in global health are far more dire than in commercial innovation, which might be limited to loss of private investment. In global health, failed interventions can result in loss of life, wellbeing, and funds that might have otherwise been used for proven interventions. In this way, global health innovators might be better served by approaches to design that encourage iterative testing for efficacy and safety and grounded in local expertise. The TB Tech project seemed to mostly benefit from the HCD approach in these ways, resulting in an innovation that was ready to be embraced by users and stakeholders and required only minor adjustments to providers' already strained workflows. Still, only further implementation and research using HCD and other approaches to design can provide adequate evidence about how to select the best approach for a given global health challenge.

## Conclusions

The TB Tech initiative provides evidence that human-centered design can facilitate digital innovation in resource-constrained settings. Using this approach, our team improved our understanding of the needs and assets of providers in a low-resource HIV care context, created a TB clinical decision support system to improve intensive case-finding and IPT initiation among patients living with HIV, and implemented the system among 3 pilot sites and then an extensive network of 68 HIV clinics in Western Kenya. As leaders of HIV programs worldwide introduce innovative digital solutions, techniques in human-centered design can facilitate the process of developing and using mHealth and eHealth tools to address complex problems.
